# Michael Chopp, Ph.D., Distinguished Professor of Physics at Oakland University, Michigan, United States: Adapted Interview from the 12^th^ World Congress for NeuroRehabilitation (WCNR), Vienna, 2022

**DOI:** 10.25122/jml-2023-1008

**Published:** 2023-02

**Authors:** Andreea Doria Constantinescu, Alexandra Gherman

**Affiliations:** 1 RoNeuro Institute for Neurological Research and Diagnostic, Cluj-Napoca, Romania

**Interviewer: Andreea Doria Constantinescu**

**Interviewee: Professor Michael Chopp**

Michael Chopp, Ph.D., FAHA, FESO, is Vice Chairman for Research of the Department of Neurology, Scientific Director of the Henry Ford Neuroscience Institute, and the Zoltan J. Kovacs Chair in Neuroscience Research at Henry Ford Health.

He received his MS and doctorate degrees in Mathematical and Solid-State Physics from New York University. After nearly 10 years of working as a Physicist and as a Professor of Physics, Dr. Chopp turned his interest to translational research in neuroscience.

Professor Chopp’s research has primarily focused on:

- Cellular and molecular biology of ischemic cell injury;
- Pathophysiology of stroke, traumatic brain injury, vascular dementia, peripheral neuropathy, multiple sclerosis, and cancers;
- Combination thrombolytic and neuro and vascular protective therapies for stroke;
- Mechanisms of neuroprotection and neurovascular restoration;
- Cell-based and pharmacological neurorestorative therapies for stroke, traumatic brain injury and neurodegenerative disease;
- Molecular and cellular mechanisms underlying neurovascular remodelling and the induction of brain plasticity leading to functional and behavioural recovery after neural injury;
- Treatment of glioma, ovarian, prostate, and breast cancers;
- Exosomes/microRNA for the treatment of neurological injury and disease;
- Magnetic resonance imaging.

Dr. Chopp also has more than 780 peer-reviewed publications, wrote more than 50 book chapters (H index=160, >96,000 citations), delivered >500 plenary lectures, and invited presentations. He has also received many awards for his research. He has chaired National Institutes of Health (NIH) study sections and has served as a consultant to government agencies, the U.S. National Institutes of Health, and the pharmaceutical industry.

**A.D.C.:** Dear Professor Michael Chopp, we are here, in Vienna, for the 12^th^ World Congress for NeuroRehabilitation, organised by the World Federation for NeuroRehabilitation. What is your first-hand opinion on the event so far?

**M.C.:** It is wonderful having people from all over the world coming together, sharing their ideas, people from multiple disciplines, specialists in physical therapy, neurologists, and neurorehabilitation gathering with the unified goal of improving patient outcome post neural injury. This multi-disciplinary gathering creates a synergy, a synergy of knowledge, growth, and opportunity for really advancing the field.

**A.D.C.:** What is the impact of such events on the new generation specialists in neurorehabilitation?

**M.C.:** New generations learn from old generations, both from mistakes and advances. We must build on the knowledge we have and, importantly to constantly challenge the knowledge we have, to advance the field in order to improve patient care. So, growth in any field requires analysis of what is known, what is missing, and how we question and advance what we know. To advance a field, we must understand what is needed and what is missing, what the patient needs, and what we are not fulfilling in our professional responsibilities and applications.

**A.D.C.:** From your perspective, what is the role of hybrid multidisciplinary events in developing rehabilitation research and practice?

**M.C.:** I think the nature of rehabilitation intrinsically has to be a multidisciplinary approach. The patient has an injury, the patient has a stroke, the patient has a traumatic brain injury; what we must do is treat the entire patient, not just the injury. What we have to do is understand how the injury affects other organs, how it affects the emotional status and the psychological status of the patient; since we are dealing with the totality of the human being in response to an initiating injury, a multidisciplinary and an integrative therapeutic approach is required.

**A.D.C.:** What are the most crucial factors that clinicians should consider in stroke neurorehabilitation?

**M.C.:** I think the most crucial factor is not to be siloed, not to be just focused on the injury. We have to realise that when there is an injury, every aspect of that human being has been challenged and potentially compromised. The work I have presented at this meeting demonstrates that when you have a neural injury, you impact systemic vasculature and other organs, you impact cardiac function, liver function, what happens in the gut, all interacting in a way that determines how that patient will respond to an injury. It is critically important for us to have input and to treat all aspects of that injury: the emotional and the secondary events – we showed, for instance, that cognitive dysfunction post-neural injury, let’s say, after a stroke, is directly related to cardiac function, so we need someone not only to treat the injury to the brain but to address secondary cardiac dysfunction, to look at systemic effects and see whether the treatment of the secondary events can impact the outcome of the patient.

**A.D.C.:** Thank you! How vital is pharmacological support in the stroke neurorehabilitation process, and what evidence is available?

**M.C.:** I think the idea of ‘pharmacologic’ has to be expanded. It is not simply a ‘drug’. And there is no single magic bullet that I think will determine what will benefit the patient. First of all, ‘pharmacologic’ implies a drug, and I think we have shown, and many others have also demonstrated that we may need multimodal biological-based therapeutics, which are far more intricate and complex than a single pathway-altering pharmacologics. Years ago, we initiated the concept of ‘stem cell therapies for recovery’. Now, we have advanced that concept into using biological nanoparticles for the treatment of neurological injury, to enhance neurovascular recovery and thereby to improve recovery post-neural injury. I think ‘pharmacologic’ – the term, is a bit too narrow in that there are also biologics that are now rapidly emerging that will have a great therapeutic impact on the treatment of the patient. Also, I think we must consider using an agent – whether it is a pharmacologic or a biologic, in combination with physical therapy and other therapeutic modalities. We have to integrate many therapeutic approaches to enhance the outcome – neurologic, cognitive, and functional outcomes of the injured patient.

**A.D.C.:** Thank you very much for accepting our invitation for this interview and for sharing a little of your experience and your valuable opinions with us!

**M.C.:** I am delighted to be here, I am so fortunate – after Covid, to be with my dear colleagues and to see such warmth and enthusiasm! I think everyone feels this. It’s sparkling, the experience of being at this congress now and being with my colleagues!

**A.D.C.:** Thank you very much!

**M.C.:** Thank you!

